# The social ecology of water in a Mumbai slum: failures in water quality, quantity, and reliability

**DOI:** 10.1186/1471-2458-13-173

**Published:** 2013-02-26

**Authors:** Ramnath Subbaraman, Shrutika Shitole, Tejal Shitole, Kiran Sawant, Jennifer O’Brien, David E Bloom, Anita Patil-Deshmukh

**Affiliations:** 1 Partners for Urban Knowledge, Action, and Research (PUKAR), Mumbai, Maharashtra, India; 2 Division of Infectious Diseases, Brigham and Women’s Hospital, Boston, MA, USA; 3 Department of Global Health and Population, Harvard School of Public Health, Boston, MA, USA

**Keywords:** Diarrhea, Water quality, Water quantity, Contamination, Coliform bacteria, *E. coli*, Household water treatment, Safe storage, Urban slums, India

## Abstract

**Background:**

Urban slums in developing countries that are not recognized by the government often lack legal access to municipal water supplies. This results in the creation of insecure “informal” water distribution systems (i.e., community-run or private systems outside of the government’s purview) that may increase water-borne disease risk. We evaluate an informal water distribution system in a slum in Mumbai, India using commonly accepted health and social equity indicators. We also identify predictors of bacterial contamination of drinking water using logistic regression analysis.

**Methods:**

Data were collected through two studies: the 2008 Baseline Needs Assessment survey of 959 households and the 2011 Seasonal Water Assessment, in which 229 samples were collected for water quality testing over three seasons. Water samples were collected in each season from the following points along the distribution system: motors that directly tap the municipal supply (i.e., “point-of-source” water), hoses going to slum lanes, and storage and drinking water containers from 21 households.

**Results:**

Depending on season, households spend an average of 52 to 206 times more than the standard municipal charge of Indian rupees 2.25 (US dollars 0.04) per 1000 liters for water, and, in some seasons, 95% use less than the WHO-recommended minimum of 50 liters per capita per day. During the monsoon season, 50% of point-of-source water samples were contaminated. Despite a lack of point-of-source water contamination in other seasons, stored drinking water was contaminated in all seasons, with rates as high as 43% for *E. coli* and 76% for coliform bacteria. In the multivariate logistic regression analysis, monsoon and summer seasons were associated with significantly increased odds of drinking water contamination.

**Conclusions:**

Our findings reveal severe deficiencies in water-related health and social equity indicators. All bacterial contamination of drinking water occurred due to post-source contamination during storage in the household, except during the monsoon season, when there was some point-of-source water contamination. This suggests that safe storage and household water treatment interventions may improve water quality in slums. Problems of exorbitant expense, inadequate quantity, and poor point-of-source quality can only be remedied by providing unrecognized slums with equitable access to municipal water supplies.

## Background

In 2004, the World Health Organization (WHO) and UNICEF estimated that 961 million urban dwellers worldwide must gain access to an improved water supply by 2015 to achieve the Millennium Development Goal of halving the proportion of people without access to safe water [1]. Water insecurity is associated with adverse health outcomes, especially in urban slum communities [2]. For example, multiple studies of urban slums in Africa and South Asia have found diarrhea to be one of the top two causes of morbidity and mortality for children under five [3-7].

As of 2011, India is estimated to have a slum population of approximately 93 million people [8]. Approximately 50% of these slums are “notified,” or recognized by the government, while the remaining slums are unrecognized, or “non-notified” [9]. In Mumbai, as in many other Indian cities, only notified slums are ensured security of residential tenure and access to water, sanitation, and electricity [10]. In non-notified slums, where the government does not provide water access, complex informal systems of water procurement, distribution, and storage emerge so that residents can access this basic amenity. By “informal,” we refer to community-created or private systems that are outside of the purview of government regulation. While they are rarely the subject of research, these informal distribution systems may adversely affect health and social equity due to various insecurities, including provision of inadequate water quantity, unpredictability of water access, and high costs. In particular, understanding the impact that such informal systems have on bacterial contamination may assist in designing appropriate water safety interventions in these communities.

In this paper, we evaluate an informal water distribution system in Kaula Bandar (KB), a non-notified slum in Mumbai, using the following commonly accepted health and social equity indicators: cost of water, quantity of water consumed at the household level, microbiological and chemical quality of water, and residents’ opinions of hardships associated with water access [2]. For each major indicator, we identified critical research questions that formed the basis for our investigation of the informal water distribution system (Table 1). We were particularly interested in identifying predictors of bacterial contamination of drinking water and of quantity of water consumed at the household level, given the direct association of these two indicators with health outcomes. The data presented here were collected in two different studies: the 2008 KB Baseline Needs Assessment (BNA) and the 2011 KB Seasonal Water Assessment (SWA).

**Table 1:** Water-related indicators and key research questions when evaluating the informal water distribution system in the urban slum of Kaula Bandar

**Indicator**	**Research questions**	**Metrics**
Quality	• What percentage of drinking water is contaminated with bacteria by the time it reaches the point of consumption?	• Total coliform bacteria and E. coli levels measured in numerous water samples
	• Given the complexity of the informal distribution system, where along the chain of access does water get contaminated with bacteria, if at all? Specifically, does most contamination occur at the level of the motorized pumps (the point-of-source), the hoses (the distribution network), or the household drinking water storage containers (the point-of-use)?	
	• What are the key predictors of bacterial contamination of drinking water? Specifically, what are the roles of season, frequency of refilling water, quantity of water consumed, etc., on contamination?	• Gross appearance of water, water treatment method used, gross appearance of storage container, composition of storage container, and days since container was last filled and cleaned for every water sample collected
Quantity	• What percentage of households fail to achieve the WHO minimum recommendation of 50 liters per capita per day (l/c/d) for quantity of water consumption?	• Quantity of water used in the last week by each household represented in liters per capita per day (l/c/d)
	• What percentage of households fail to achieve a consumption threshold of 20 l/c/d, which is associated with high risk to health?	
	• What are the key predictors of use of an inadequate quantity of water? Specifically, what are the roles of season, cost of water, and total money spent on purchasing water?	
Cost	• What is the average cost that residents pay per 1000 liters of water?	• Money spent by each household on purchasing water in the last month and week
	• How does the cost of water obtained through the informal distribution system compare to the cost paid by residents of other notified (government-recognized) slums who obtain water through the formal municipal system?	
	• What percentage of monthly household income is spent on purchasing water?	• Mean household income in the community obtained from a separate survey of 521 randomly selected households
Reliability	• What are the health and economic consequences of an unreliable water distribution system? Specifically, how does periodic “system failure” of the informal distribution system impact key indicators such as quality, quantity, and cost?	• Data on major water indicators specifically collected from study households during an episode of “system failure”

## Methods

### Study site

KB is a non-notified slum of approximately 10,000 people located on a wharf on Mumbai’s eastern waterfront. Like other slums, living spaces consist of shanties built of corrugated metal, wood, and cement. Only 3% of homes have a latrine inside. As a result, the vast majority of children and 14% of adults engage in open defecation, while the remainder of adults use a handful of pay-for-use toilets in the community or travel long-distances to access pay-for-use toilets outside of the slum [11]. KB is located on central (federal) government property, which precludes it from receiving services provided by the city government, such as water and electricity [10]. Nearly all KB residents therefore purchase water via an informal distribution system run by private vendors.

### The informal water distribution system in KB

The sources of KB’s water supply are two underground pipes that were installed decades ago by the fire department for emergency use. Water vendors, nearly all of whom are residents of KB, have created entry points in the fire brigade pipes from which water is extracted using motorized pumps. Water is then pumped through rubber hoses that extend hundreds of meters to reach community lanes. Given the absence of space on the main road of the community and the lack of underground piping infrastructure, the hoses travel through the surrounding ocean and trash dumps and are often visibly compromised by holes. Tracing each vendor’s water distribution area (i.e., the households receiving water from a particular vendor’s motor and hose system) formed the basis for sample selection in the SWA, as is described further below.

**Figure 1:**
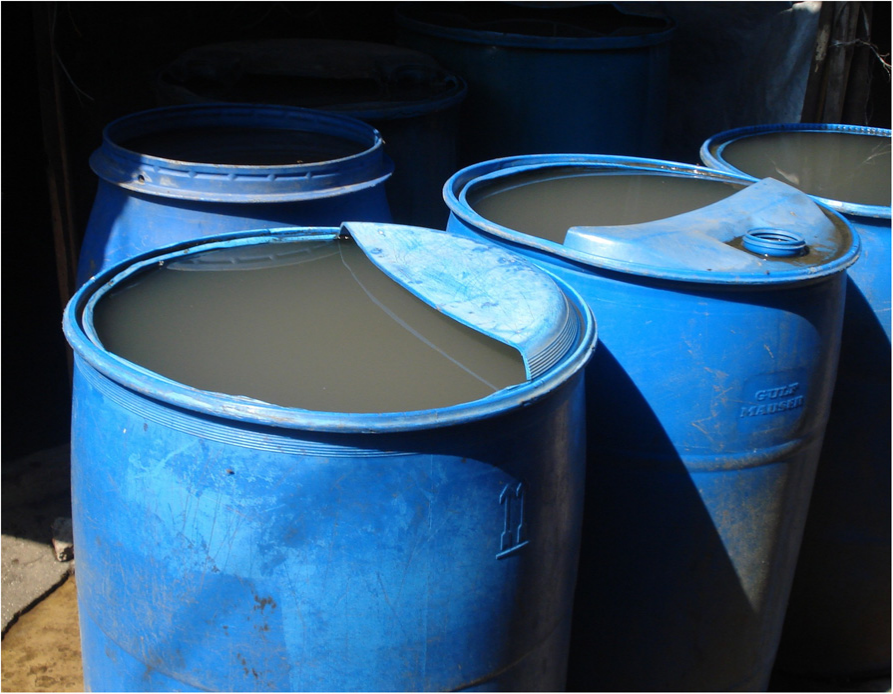
**Large containers for holding “storage water”.** Legend: Large 300-liter plastic drums commonly placed outside of the home to hold “storage water.” This water is used for bathing, toileting, and washing clothes (non-drinking purposes).

**Figure 2:**
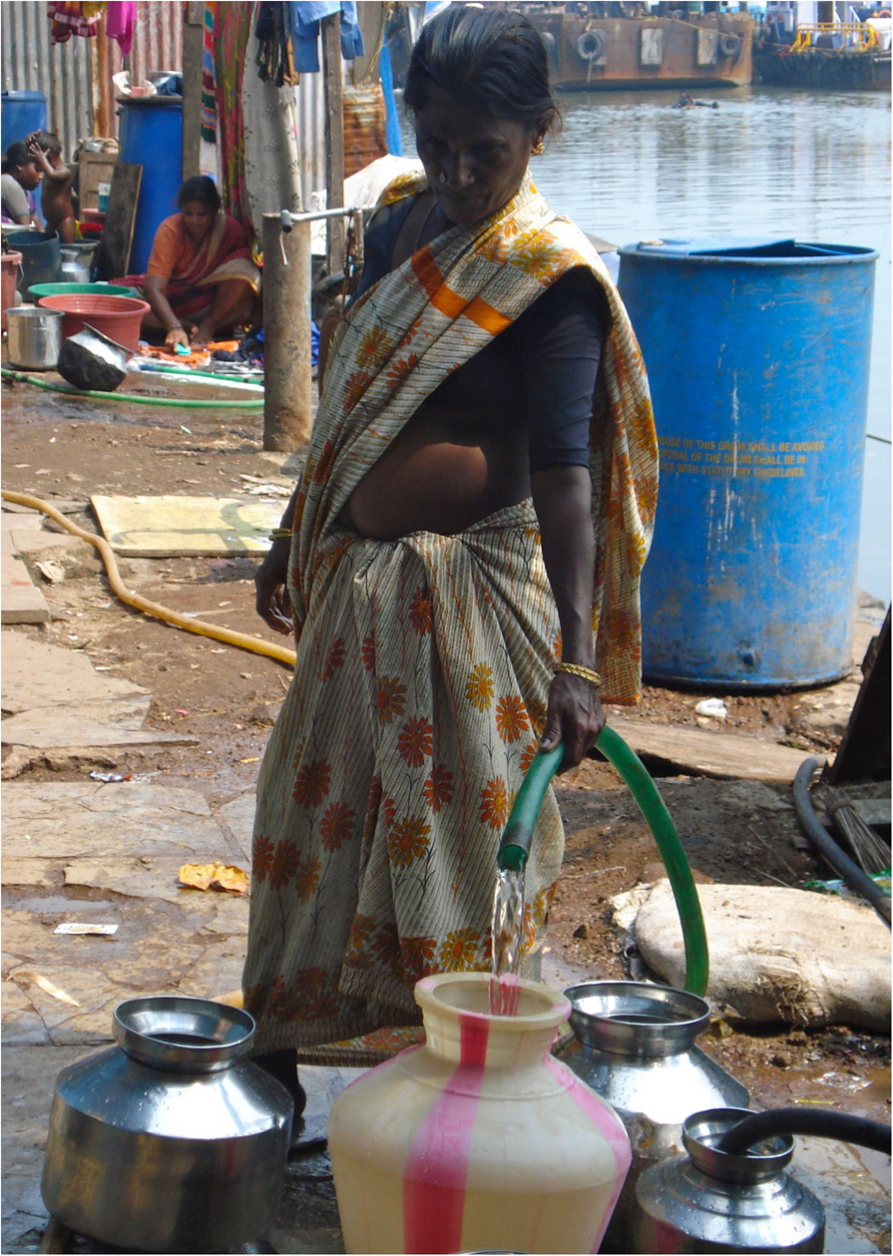
**Small containers for holding “drinking water”.** Legend: A woman in Kaula Bandar fills smaller metal and plastic containers used for storing drinking water. Nearly all drinking water containers used in the community are wide-mouthed, allowing for contamination of water by people’s hands.

Within each household, water is stored in two types of containers. Large plastic drums with capacities of 100 to 300 liters are placed outside the home and hold “storage water,” which is used for bathing, toileting, and washing clothes (Figure 1). Water from these larger storage containers is used for drinking only during times of severe water scarcity. Smaller containers with a capacity of one to 50 liters are kept inside the home and are used to store “drinking water” (Figure 2). Notably, nearly all of the drinking water containers in KB are wide-mouthed and allow people to directly access water from the containers with their hands, a detail that has major implications for household-level water contamination.

KB’s informal water distribution system is vulnerable to widespread failure. Local officials (from the central government agency on whose land KB is situated) occasionally raid and confiscate motors tapping into the fire brigade pipes, cutting off water access to KB’s residents. Such episodes of “system failure” occur a few times a year. When this happens, most KB residents roll large storage drums at least one kilometer, and as far as two kilometers, to access taps in the next closest community, while others get water from private tankers.

The water vendors who run the informal water distribution system incur significant costs to maintain the system, all of which are passed on to community residents who purchase this water. For example, based on interviews with multiple water sellers, we estimated that they pay Indian rupees (INR) 15,000 to 20,000 for a new motorized pump (used for extracting water from underground pipes), which is US dollars (USD) 273 to 364. A used motorized pump costs INR 10,000 to 12,000 (USD 182 to 218); however, these require frequent repair every few months, which generally costs INR 500 (USD 9) per repair. When local officials confiscate motorized pumps every few months, water vendors pay bribes of INR 500 to 1000 (USD 9 to 18) to get motorized pumps back, though they are often unable to get the pumps back and are forced to buy new ones. The rubber hoses used to distribute water to community lanes cost INR 5000 (USD 91) per 100 meters, and a few hundred meters of hose are often required to provide water to community lanes from each motorized pump. Finally, each water vendor usually hires one or two other people to facilitate water distribution, and these individuals are remunerated by receiving free water.

### Study design for the 2008 Baseline Needs Assessment (BNA)

The BNA is a 92-question household survey covering five domains: income and assets, access to government benefits, access to education, access to water and sanitation, and common causes of morbidity. Only the water-related data are reported here. The survey was implemented from August to December 2008 by a group of 15 uniformly trained interviewers. The questionnaire was administered to a head of household greater than 18 years of age after receiving written informed consent. Since men in KB work long hours outside of the home, 71% of respondents were women.

Researchers coded every living space in KB. Every occupied household was requested to answer the questionnaire at least once during the study period. Due to migrancy, many households were empty and locked at any given time. Out of 2,439 total living spaces, 900 were permanently locked with no identifiable occupants. Of the remaining 1539 occupied households, 959 responded to the survey, yielding an estimated 37.7% non-response rate. This relatively high non-response rate is attributable to the long work days of many adults, who return home at hours when the community is not safely accessible to researchers.

### Study design for the 2011 Seasonal Water Assessment (SWA)

The SWA evaluated microbiological and chemical quality of water, quantity of water used, and cost of water during four study periods: winter (February 2011), summer (early May 2011), monsoon (August 2011), and an episode of “system failure” in late May 2011 (see above), when KB residents had to seek out alternative water sources. This seasonal design originated from findings of prior studies [12,13] and from observations by researchers of possible variations in household water availability and cross-contamination risk during Mumbai’s different seasons.

**Figure 3:**
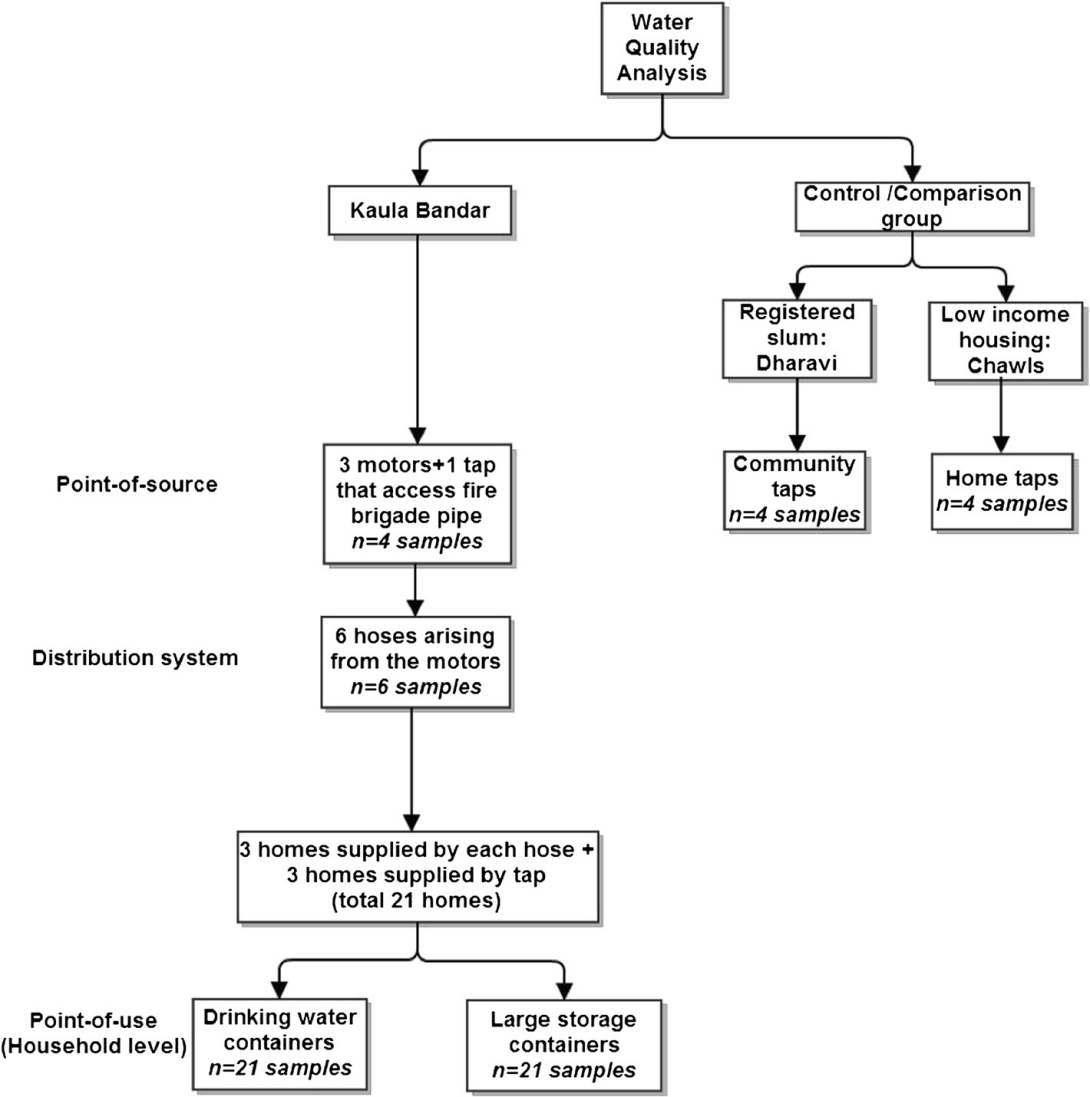
Water quality sampling strategy for winter, summer, and monsoon seasons during the Seasonal Water Assessment.

Researchers uniformly trained in sterile technique collected water samples in autoclaved bottles from many points along the informal distribution network. These points included: three motorized pumps that extract water from fire brigade pipes (i.e., the “point-of-source”), six hoses that transport water from the three pumps to lanes in KB (i.e., the “distribution network”), and three randomly selected households supplied with water by each of the six hoses (i.e., the “point-of-use”). An additional point-of-source sample was collected from a household with a tap that directly accesses a fire brigade pipe. Since some households buy water directly from this tap (i.e., not through hoses), three such households were also selected for water collection, bringing the total household sample size to 21 (Figure 3). The same 21 households were followed for all four periods of data collection.

**Figure 4:**
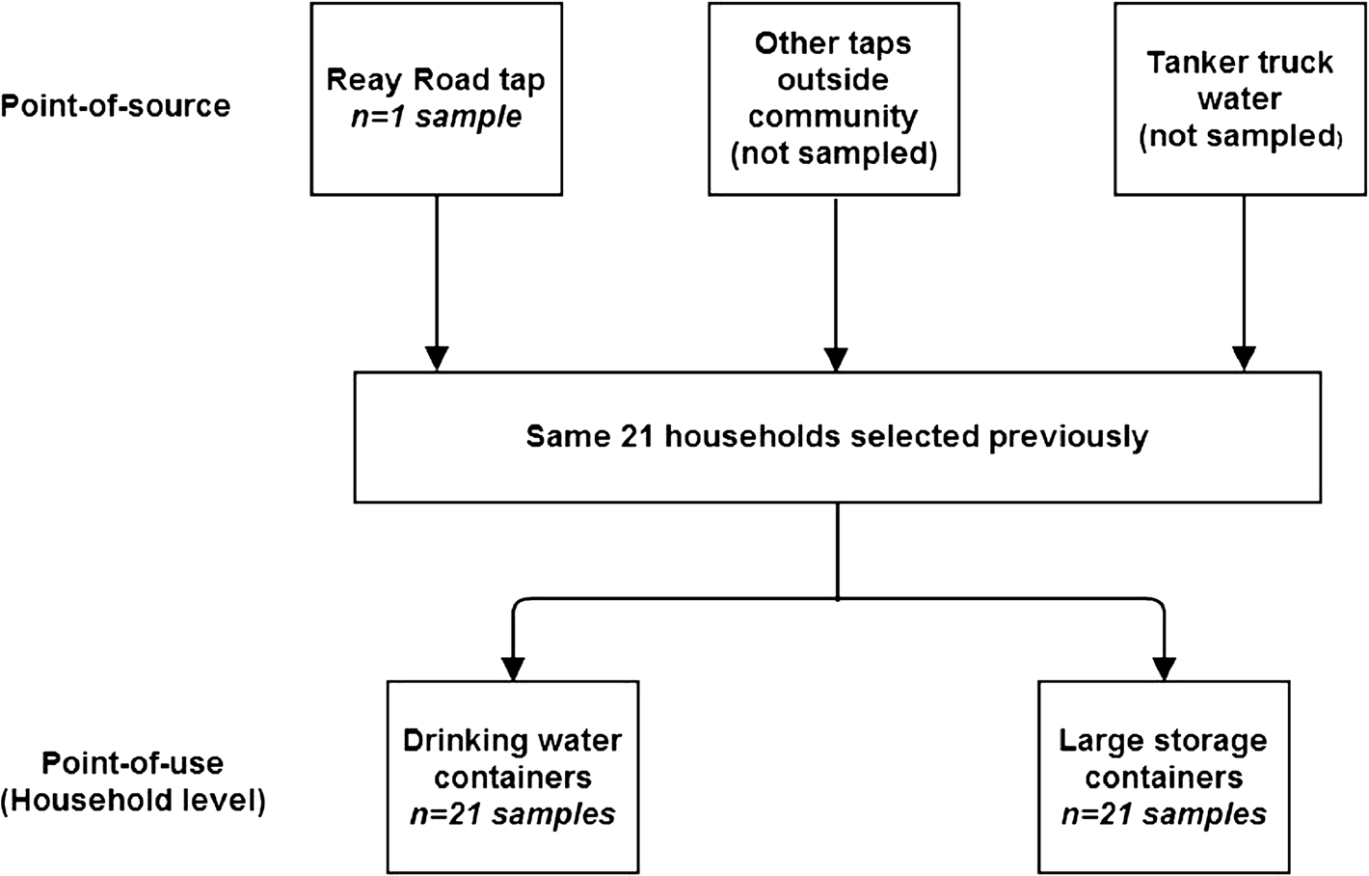
Water quality sampling strategy for the episode of “system failure” in the Seasonal Water Assessment.

For comparison to KB point-of-source samples, control point-of-source tap water samples were obtained from chawls (low-income housing) and from Dharavi, a notified slum with legal water access (Figure 3). During “system failure,” a point-of-source sample was collected from the Reay Road tap, the main tap outside of KB used by residents when the water distribution system fails (Figure 4). Given environmental exposure to seawater in KB, two ocean water samples were also collected for testing in each season.

All 229 water samples were transported to Equinox Labs, which is certified by the International Organization for Standardization (ISO 9001:2008) and the National Accreditation Board for Laboratories (NABL), within four hours of collection. Equinox Labs performs external quality testing by cross-checking multiple samples with five other NABL certified labs at least once a year, and internal quality checks for water testing are carried out on a daily basis. Samples were tested for total coliform counts, *E. coli*, and chemical parameters, as per protocols recommended by the American Public Health Association and the Indian Standards Chemical Division Council [14].

The chemical parameters tested included pH (normal 6.5-8.5), total dissolved solids (normal <500 mg/L), turbidity (normal <5 NTUs), total hardness (normal <300 mg/L), calcium (normal <75 mg/L), magnesium (normal <30 mg/L), and sulphates (normal <200 mg/L). Given the fact that water from the motorized pumps and distribution hoses in KB is at risk for contamination with ocean water, we also tested these samples in the winter season for salinity (normal <0.8 dS/m). Microbiological testing was performed as follows: 100 mL of each sample were filtered through a 0.22-micron, 47 mm diameter membrane using a vacuum pump. The filter was subsequently transferred to an m-Endo agar plate for culturing. Samples were incubated at 35**°**C for 24 hours, at which time coliform bacterial colonies were counted using an automated colony counter. *E. coli* was reported as being present or absent after interpretation of indole, methyl red, Voges-Proskauer, and citrate tests.

In addition to water sample testing, in all four study periods, researchers administered questionnaires to an adult older than age 18 in each of the 21 households. Since questionnaires were administered around the time of water collection, they were usually administered to the adults who most commonly interact with water vendors and who therefore were most likely to provide accurate information on water spending. The questionnaire assessed common health and social equity indicators, specifically monthly and weekly water charges and quantity of water used in the last week [2]. For each household water sample collected, it also assessed the following parameters possibly associated with contamination, which were selected based on a review of prior studies [15-17]: gross appearance of the water, composition of the water container, and length of time since the container was last cleaned and filled with water.

Quantity of water used was assessed through a detailed inventory of every drinking and storage water container in each household. Prior to starting data collection in the field, researchers were uniformly trained to recognize the volume of water held by containers of various sizes and shapes commonly used to store water in the community. In each household surveyed, researchers carefully estimated the capacity in liters of each container and the number of times it had been filled in the last week. These numbers were tabulated to estimate the total amount water used in the last week by each household.

Multivariate logistic regressions were performed to understand associations between microbiological water quality (i.e., the presence or absence of coliforms and *E. coli*) and other drinking water and container characteristics (Table 2). Multivariate linear regressions were performed to understand associations between quantity of water consumed and season and cost variables (Table 2). Statistically significant variables (p < 0.05) were included in the multivariate models using the forward stepwise method, and the best reduced model was built based on a −2 log likelihood value. Statistical analyses were performed using SPSS (version 19; Chicago, IL, USA).

**Table 2:** Variables included in multivariate regression analyses

**Analysis of predictors of coliform or *E. coli *contamination of drinking water**	**Analysis of predictors of quantity of water used in the household**
Season	Season
Quantity of water consumed (in l/c/d)	Total money spent purchasing water in the last week (in INR)
Water treatment method used	Cost of water (in INR per 1000 liters)
Gross appearance of water sample (i.e., clear or cloudy)	
Gross appearance of container cleanliness (i.e., clean or dirty)	
Container material (i.e., metal, plastic, or clay)	
Days since the container was last filled	
Days since the container was last cleaned	

Research protocols were approved by the Partners for Urban Knowledge, Action, and Research (PUKAR) Institutional Ethics Committee, which is registered under federal-wide assurance number FWA00016911.

## Results

We summarize findings from the BNA and SWA according to the following categories: water costs and hardships, water quantity, water quality and storage, environmental exposures, and residents’ opinions on water access.

### Water costs and hardships

Table 3 presents SWA data on household water costs during the four study periods. A majority of households paid a monthly base fee to water vendors of Indian rupees (INR) 150 to 400 per month, which is approximately US dollars (USD) 2.73 to 7.27, for water during all study periods. In summer and during episodes of system failure, most households pay additional weekly fees to acquire extra water. Based on monthly and weekly water costs, as well as water quantity data, we were able to estimate the cost spent per 1000 liters of water for each household. By comparing these costs to the standard government water charge in 2011 of INR 2.25 (USD 0.04) per 1000 liters, we estimate that KB residents spend 52 to 206 times more than residents of slums with legal water access, depending on the season (Table 3).

**Table 3:** Water indicators from the 2011 seasonal water assessment

**Water indicator**	**Study period**			
	**Winter**	**Summer**	**Monsoon**	**System failure**^**4**^
**Water costs**				
Monthly spending on water in INR^1,2^	379.8 (139.8)	1022.0 (676.6)	378.6 (114.1)	--
*Mean (SD)*				
Monthly spending on water in USD^2,3^	6.91 (2.54)	18.58 (12.30)	6.88 (2.07)	--
*Mean (SD)*				
Monthly spending on water as a percentage of the mean household income in KB^2^	5.9%	15.9%	5.9%	--
Estimated cost in INR per 1000 liters of water^2^	145.4 (87.0)	327.9 (258.9)	117.9 (56.6)	463.1 (297.2)
*Mean (SD)*				
Estimated cost in USD per 1000 liters of water^2^	2.64 (1.58)	5.96 (4.71)	2.14 (1.03)	8.42 (5.40)
*Mean (SD)*				
Comparison to government rate of INR 2.25 per 1000 liters of water^2^	65	146	52	206
*Number of times more expensive*				
**Quantity of household water use**				
Liters per capita per day of water use	22.6 (12.6)	31.2 (23.6)	25.6 (13.2)	23.8 (14.2)
*Mean (SD)*				
Households using <50 liters per capita per day	20 (95.2)	17 (80.95)	19 (90.4)	20 (95.2)
*n (%)*				
Households using <20 liters per capita per day	9 (42.9)	8 (38.1)	8 (38.1)	10 (47.6)
*n (%)*				

^1^INR = Indian rupees.

^2^These figures are based on both the standard monthly payment to water vendors *and* additional weekly payments given during summer season and during periods of system failure.

^3^USD = US dollars.

^4^Data for monthly spending on water during the episode of system failure are not presented here, because the monthly costs are the same as those for the summer season, since the system breakdown happened in the same month. However, using the monthly summer costs plus the extra weekly cost spent during the period of system failure, we are able to present the estimated cost per 1000 liters of water for the system failure episode.

Based on income data for 521 households from another study (PUKAR's 2012 mental health study in KB), we estimate the mean monthly household income in KB to be INR 6411 (USD 116.56) [18]. Using this figure, we estimate that KB residents spend 5.9% to 15.9% of their monthly household income on buying water in different seasons (Table 3). We then estimated the mean total cost of water spent by a household over an entire year to be INR 6479 (USD 117.80), by assuming that winter season lasts from October to February, summer season from March to May, and monsoon season from June to September. Based on this figure, KB households spend approximately 8.4% of their yearly income on water.

A 2010 PUKAR census of KB found a population of approximately 10,000 people and an average household size of 4.8 people. Using these figures, we calculated the approximate yearly amount spent on water by the entire community to be INR 13,498,083 (USD 245,420.00). We compared this cost to an ideal scenario in which all 10,000 residents receive the WHO-recommended minimum of 50 liters per capita per day (l/c/d) of water for every day of the year at the standard government charge of INR 2.25 (USD 0.04) per 1000 liters. In such an ideal scenario, the entire community would spend INR 410,625 (USD 7,466.00) on water yearly.

This suggests that residents spend an excess amount of INR 13,087,458 (USD 237,954.00) yearly on water in the current informal system. We compared this excess amount to the cost of placing comprehensive water infrastructure in KB (in the form of a new pipeline with community taps), which is INR 2,500,000, or approximately USD 45,455.00 (personal communication from Ward Corporator Mangesh Bansod). Based on this figure, the excess amount spent on water under the informal system could pay for entirely new water infrastructure in KB more than five times every year.

The BNA provides additional data on water-related hardships. In the BNA, 952 households (99.3%) report having to regularly purchase water. The majority, 529 (55.2%), are only able to access water every three or more days (Table 4). Most households, 817 (85.2%), have water delivered via water vendors’ hoses, while 125 (13.1%) must fetch water from outside of their lanes. Due to queues at hoses or time involved in fetching water, 370 (38.5%) spend > ½ hour on obtaining water. Many households report a subjective perception that the local water situation affects their family members’ health, ability to go to work, and ability to go to school (Table 4).

**Table 4:** Water-related data the baseline needs assessment

	**n (%)**
Frequency of water access	
Does not purchase water	7 (0.7)
Daily	144 (15)
Every two days	279 (29.1)
Every three days	231 (24.1)
Every four days	236 (24.6)
Weekly	62 (6.5)
Time spent obtaining water	
<½ hour	584 (60.9)
½ hour to 1 hour	291 (30.3)
1 hour to 1 ½ hours	65 (6.8)
More than 1 ½ hours	14 (1.4)
Mode of obtaining water	
Delivery via water vendors’ hoses	817 (85.2)
Fetch water from outside their lanes	125 (13.1)
Other	17 (1.7)
Does lack of water affect you or your family members’:	
Health?	860 (89.7)
Ability to go to work?	371 (38.7)
Ability to go to school?	87 (9.1)
Ability to study?	38 (4.0)
Ability to start a new business?	13 (1.4)
Ability to increase productivity in your current business?	14 (1.5)
Water purification methods used^1^	
Filter-based water purifier set	5 (0.5)
Cloth filter used during collection	274 (25.8)
Boiling	165 (17.2)
Alum	8 (0.8)
Other purifying agents (i.e., chlorine)	18 (1.9)
No purification method used	568 (59.2)
Why do you think that the community lacks running water?^1^	
Land belongs to an external agency, so that municipal government cannot provide water	426 (44.4)
The community is unauthorized	111 (11.6)
No one cares about the community	182 (19.0)
Don’t know	289 (30.1)
Other	18 (1.9)
Who has the primary responsibility for providing water to the community?^1^	
The local politician	390 (40.7)
The municipal system	320 (33.4)
Residents themselves	44 (4.6)
Other (i.e., local water vendors)	263 (27.4)

^1^These questions allowed respondents to give multiple answers to the questions, so the percentages add up to more than 100%.

### Quantity of water consumed at the household level

**Figure 5:**
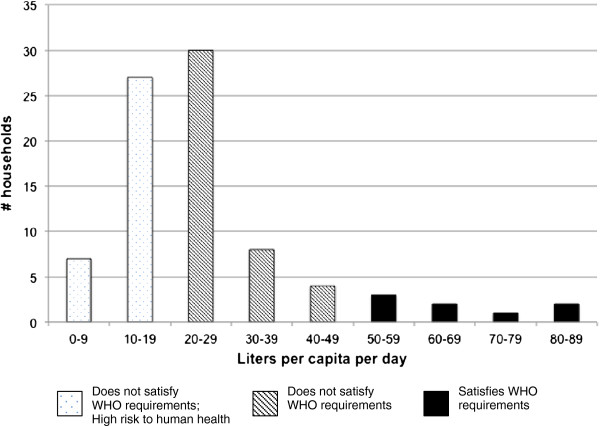
Histogram of water quantity data from all study periods of the Seasonal Water Assessment.

Data from the SWA show that, in different seasons, 81-95% of households do not meet the WHO recommendation that all human beings use a minimum of 50 liters per capita per day (l/c/d) of water (Table 3 and Figure 5) [19]. A significant percentage use less than 20 l/c/d, a consumption level associated with a “high” or “very high” level of health concern per the WHO [19]. In the multivariate linear regression model (R^2^ = 0.329), total money spent on purchasing water was positively associated with the quantity of water consumed (β-coefficient = 0.569, p < 0.001), while the cost of water in INR per 1000 liters was negatively associated with the quantity of water consumed (β-coefficient = −0.691, p < 0.001).

### Water quality and storage

According to the BNA, most households, 568 (59.2%), do not use any method of water purification, while 25.8% use a cloth filter and 17.2% boil their drinking water prior to consumption (Table 4). Of note, our informal observations suggest that many adults also boil water prior to consumption in beverages such as tea or coffee; however, the majority of water is consumed “raw” without being boiled in beverages, especially for children, who are most likely to suffer from diarrheal illness. The SWA similarly found that 12 households (57.1%) did not use any method of water purification during any of the study periods. In the SWA, 35.7% of drinking water containers were made of plastic, 60.7% of metal, and 3.6% of clay. One hundred percent of storage water containers were made of plastic.

Table 5 shows the SWA’s microbiological testing results. The monsoon season had a different pattern of water contamination as compared to the pattern in the winter season, the summer season, or during the episode of “system failure.” We will first discuss the pattern of contamination in the winter and summer seasons.

**Table 5:** Water contamination data from the seasonal water assessment

**Water contamination indicator**	**Study period**			
	**Winter**	**Summer**	**Monsoon**	**System failure**
**Tap water samples (“point-of-source” water)**				
Chawl taps^1^ (n = 4)				
Samples with coliforms	0 (0)	0 (0)	1 (25)	--
*n (%)*				
Samples with *E. coli*	0 (0)	0 (0)	1 (25)	--
*n (%)*				
Dharavi taps^2^ (n = 4)				
Samples with coliforms	0 (0)	0 (0)	0 (0)	--
*n (%)*				
Samples with *E. coli*	0 (0)	0 (0)	0 (0)	--
*n (%)*				
Kaula Bandar motors and tap (n = 4)				
Samples with coliforms	0 (0)	0 (0)	2 (50)	--
*n (%)*				
Samples with *E. coli*	0 (0)	0 (0)	0 (0)	--
*n (%)*				
Reay Road tap^3^ (n = 1)				
Samples with coliforms	--	--	--	0 (0)
*n (%)*				
Samples with *E. coli*	--	--	--	0 (0)
*n (%)*				
**Kaula Bandar hose samples (“distribution network” water, n = 6)**				
Samples with coliforms	0 (0)	0 (0)	3 (50)	--
*n (%)*				
Samples with *E. coli*	0 (0)	0 (0)	1 (16.7)	--
*n (%)*				
**Kaula Bandar household level samples (“point-of-use” water)**				
Drinking Water (n = 21)				
Samples with coliforms	3 (14.3)	11 (52.4)	16 (76.2)	5 (23.8)
*n (%)*				
Coliform counts for contaminated samples in cfu/100 mL^4^	74.3 (36.5)	16.9 (8.2)	43.1 (8.5)	20.8 (9.8)
*Mean (SD)*				
Samples with *E. coli*	1 (4.8)	9 (42.9)	6 (28.6)	5 (23.8)
*n (%)*				
Storage water (n = 21)				
Samples with coliforms	7 (33.3)	10 (47.6)	15 (71.4)	8 (38.1)
*n (%)*				
Coliform counts for contaminated samples in cfu/100 mL^4^	45.7 (34.8)	19.5 (10.1)	40.6 (6.7)	21.1 (7.1)
*Mean (SD)*				
Samples with *E. coli*	3 (21.4)	7 (33.3)	4 (19.0)	8 (38.1)
*n (%)*				

^1^Control point-of-source samples from low-income housing units.

^2^Control point-of-source samples from a notified (government recognized) slum.

^3^This is the tap outside of the community most commonly used by Kaula Bandar residents during episodes of “system failure.”

^4^ cfu/100 mL = colony forming units per 100 milliliter.

During winter and summer, there was no coliform or *E. coli* contamination of any of the point-of-source samples from the two comparison groups, chawls (low-income housing) and Dharavi (a notified slum with legal water access). In winter and summer, none of the water samples collected from KB’s motorized pumps (which represent the point-of-source) or from KB’s hoses (which represent the distribution network) showed any evidence of contamination with coliforms or *E. coli*. Despite the absence of bacterial contamination of water at the point-of-source or in the distribution network of hoses in KB, there was significant contamination of drinking water and storage water at the household level (i.e., the point-of-use). For example, in the summer, 52.4% of drinking water samples were contaminated with coliform bacteria, and 42.9% were contaminated with *E. coli*. This suggests that all bacterial contamination of water during the winter and summer was happening at the household-level (i.e., the point-of-use) and *not* at point-of-source or in the distribution hoses.

During the episode of “system failure,” the pattern of water contamination was similar to the pattern in the winter and summer seasons, in that all contamination of water happened at the household level. The Reay Road tap, which represents the point-of-source for water during the episode of “system failure,” was not contaminated with coliform bacteria or *E. coli*. Again, despite receiving uncontaminated point-of-source water, 23.8% of drinking water samples were contaminated with both coliform bacteria and *E. coli*.

In contrast, during the monsoon season, we found multiple point-of-source samples to be contaminated. In the comparison groups, there was no contamination of any point-of-source samples from Dharavi, but one sample (25.0%) from the chawls was contaminated with both *E. coli* and coliforms. Two samples from KB’s motorized pumps (50.0%) were contaminated with coliforms but not *E. coli*, highlighting significant point-of-source contamination of water in KB in the monsoon season. During the monsoon, three hoses (50.0%) were contaminated with coliforms and one (16.7%) was contaminated with *E. coli*. Of note, all three of these contaminated hoses were connected to the motors in KB that showed evidence of contamination with coliform bacteria, suggesting that the water in these hoses became contaminated at the point-of-source and not secondarily as it ran through the distribution hoses. During the monsoon, at the household level, there was a very high rate of contamination of drinking water and storage water, with 76.2% of drinking water samples being contaminated with coliform bacteria. Of note, 75.0% of these contaminated drinking water samples were from households receiving water from uncontaminated distribution hoses. This suggests that, even in the monsoon, when there was significant point-of-source contamination of water, there was also superimposed household-level contaminated of stored drinking water at the point-of-use.

In the multivariate logistic regression analysis based on the variables noted in Table 2, season was the only significant predictor of coliform and *E. coli* contamination of drinking water (Table 6). Summer was significantly associated with both coliform and *E. coli* contamination, while the monsoon was significantly associated with coliform contamination only.

**Table 6:** Associations between microbiological contamination and study period (season) after multivariate logistic regression analysis

	**Coliforms**			***E. coli***		
	**Odds ratio**	**95% Confidence interval**	**p-value**	**Odds ratio**	**95% Confidence interval**	**p-value**
**Study period**			0.002			0.086
Winter	1.0	-	-	1.0	-	-
Summer	4.3	1.1 - 16.1	0.032	15.0	1.7 – 133.6	0.015
Monsoon	10.2	2.5 - 42.4	0.001	6.3	0.7 – 59.0	0.110
System failure	1.0	0.2 - 4.1	1.000	6.3	0.7 – 59.0	0.110

All of the water samples were also tested for common chemical parameters such as pH, total dissolved solids, turbidity, total hardness, calcium, magnesium, and sulphates. All samples were found to be within internationally accepted limits for all chemical parameters (normal ranges are noted above in the methods section). During winter season, we also specifically tested the point-of-source water samples (e.g., KB motors, Dharavi taps, and chawl taps) as well as the KB hose samples for salinity levels, given the risk for ocean water contamination of KB motor and hose samples. All of these samples had salinity levels of approximately 0.017 to 0.021 dS/m, which is well within the normal limit for human consumption of 0.8 dS/m.

### Environmental water exposure

In the BNA, 414 households (43.2%) reported flooding in their locality during the monsoon, while 291 (30.3%) reported flooding with waterlogging of their own homes during monsoon. Based on researchers’ observations, most flooding occurs during high tide in homes adjacent to the ocean, where most open defecation also takes place. Ocean water collected during the SWA was highly contaminated in all seasons, with 5/6 samples testing positive for *E. coli* and 6/6 testing positive for coliform bacteria.

### Residents’ opinions on water access problems

Table 4 presents BNA data regarding residents’ opinions on the causes of, and responsibility for, water access problems. Most identify complications with land ownership and the slum’s unauthorized (i.e., non-notified) status as the main reasons for lack of formal water access. Three-fourths feel that the local government (either local politicians or the municipal system) should be responsible for improving water access. Most, 528 (55.1%), are willing to pay fees to the government for a reliable water supply. If such a supply were established, 874 (91.1%) expect that it would provide water every day, and 297 (31.0%) expect that water would be provided through home taps.

## Discussion

Our findings have implications for future research and interventions that may improve water security in urban slums, especially in India where an estimated 50% of slums are non-notified communities that are frequently excluded from municipal water supplies. With regard to water quality, bacterial contamination occurred at two key points: in point-of-source water from KB pipelines during the monsoon season and in household level stored drinking water during all seasons. Household level water contamination was especially notable in the winter and summer, given the lack of point-of-source water contamination in those seasons. Even in the monsoon, when half of point-of-source water from motors was contaminated, most contaminated drinking water samples were from households that had received clean source water, suggesting superimposed household level contamination.

Our finding on the importance of household level contamination is similar to results of studies from other urban slums [15,16,20]. In KB, the vast majority of homes store drinking water in wide-mouthed containers that allow contamination when water is accessed with hands or vessels. Some studies suggest that encouraging safe storage of water in narrow-mouthed containers that minimize hand contact along with household water treatment (e.g., chlorination) may reduce diarrhea rates [21-24]. While a meta-analysis suggests significant benefits from these combined interventions [25], concerns remain regarding their scalability and possible overestimation of benefits due to publication bias [26]. If further evidence confirms that these interventions are viable and cost-effective, our data suggest they may be beneficial in urban slums.

Our data also highlight seasonal variations in contamination, with summer and monsoon having higher contamination rates than winter. The higher contamination rate in monsoon is partly attributable to point-of-source water contamination, though augmented household level contamination from ambient fecal matter due to flooding of homes is likely another major contributing factor. The higher contamination rate in summer may be due to frequent accessing of water containers due to heat-related body fluid losses.

The finding of seasonal variation in contamination rates may have practical implications for interventions. Given higher rates of vector and water-borne diseases in the monsoon (such as malaria, dengue, and leptospirosis), many municipal health departments in India, including Mumbai’s, dedicate resources in this season for public education campaigns. Public education on household water treatment could be paired with these larger campaigns in a cost-effective manner. Seasonal chlorination may be especially beneficial given the presence of point-of-source contamination during the monsoon, which is not addressable solely by switching to safe storage containers. Some studies question the sustainability of household chlorination of water, since changes in taste may decrease long-term adherence [27]. Encouraging temporary chlorination during seasons when risk is the highest may therefore be a more feasible approach.

While this study highlights household level issues, it also highlights major structural problems with KB’s core water supply that are probably more detrimental to health and social equity outcomes, given their impact on water quality, quantity and cost. Contamination of point-of-source water samples during the monsoon season is a case in point. Such point-of-source contamination likely reflects adulteration that occurs due to backflow of dirty water into the corroded fire brigade pipes that are the community’s primary water source. Since water is only provided to KB’s area for a couple of hours a day, there is no water pressure in these fire brigade pipes for most of the day. The absence of pressure in these pipes leaves them vulnerable to backflow from adjacent leaking sewer lines or from above-ground flooding, which is common during the monsoon season. One study suggests that point-of-source water contamination may be more detrimental to health than household-level contamination, since contaminated point-of-source water introduces new pathogens against which people are unlikely to have immunity [28].

In addition, the failure of the informal water distribution system to provide an adequate per capita quantity of water is another major structural problem. Use of an inadequate quantity of water may be as detrimental to health as poor water quality [29]. A considerable proportion of homes use less than 20 l/c/d, a consumption level associated with high health risk, since hygiene maintenance becomes difficult [19]. The estimated mean water consumption of 23–32 l/c/d in KB also highlights massive inequality in access, as city-wide data estimate the average water consumption in Mumbai to be 191 l/c/d [30].

Cost of water was a significant predictor of inadequate water consumption, which is not surprising given the extremely high cost of water in KB compared to the standard municipal rate. In addition, delivery of an inadequate volume of water for the population’s needs, the absence of community taps, limited water delivery timings, and long lines at water collection points may explain the low levels of water use. Periodic failures of the informal distribution system (as captured by our data collected during “system failure”) also introduce huge variability and escalation in the cost of water, as well as being a major cause of chronic stress and emotional distress [31].

Since point-of-source quality and quantity of water used are functions of these structural issues, these problems can only be remedied by providing equitable access to the municipal water supply, including a new pipeline, public water taps, and improved water provision timings. Furthermore, most remedies may provide only partial benefit unless piped water is provided directly to individual homes, as this is the only intervention that would obviate the need for in-home storage of water. Indeed, data suggest that provision of direct water connections to individual households decreases diarrheal morbidity much more than improving water quantity and access through public water taps alone [32].

The current informal distribution system exacts a massive economic toll: KB’s residents spend 8.4% of their yearly income on water, and the excess amount paid for water by the entire community could pay for comprehensive water infrastructure in the slum five times over every year. In addition to saving money for residents by lowering water costs, new water infrastructure would generate revenue for the municipality, as most residents are willing to pay for improved water access. Citywide data show that Mumbai’s operating ratio (overall operating expenses divided by revenue generated) is 0.49, suggesting that the municipality generates twice as much revenue supplying water as it spends on operating costs [30].

In summary, extending formal water infrastructure to slums such as KB would be a “win-win” situation for everyone. It would improve quality of life, reduce water costs, and improve health outcomes for slum dwellers while also decreasing waterborne disease burden in municipal hospitals, generating revenue for the municipality, and decreasing conflicts between the government and slum residents (i.e., by averting government raids on water motors). If widely applied, a policy of extending water access to non-notified slums would also facilitate meeting the Millennium Development Goal for water in urban India [1].

One limitation of this study is the relatively small sample of households chosen for the seasonal assessment of per capita water consumption and water quality. Future community-based studies with larger sample sizes may help to reinforce our findings, especially with regard to seasonal variations in water quality. In addition, our data on the economic costs borne by community residents only reflect the high cost of obtaining water under informal circumstances. The lack of an adequate quantity of uncontaminated, potable water likely exacts additional costs by greatly increasing the burden of diarrheal, upper respiratory, helminthic, and skin diseases. These diseases may take an economic toll on households by contributing to lost days of work, increased spending on medications, increased health care provider visits, and decreased productivity [33]. Indeed, while we have already highlighted a substantial economic toll on the community secondary to the informal water distribution system, if anything, our calculations are likely to be an underestimate of the overall costs.

## Conclusions

This paper highlights major failures in the quantity, quality, and reliability of water provided through an informal distribution system in a non-notified slum in Mumbai. While we have presented data from a single slum community, we would argue that this case study sheds light on similar situations faced by a significant proportion of urban dwellers in India and other cities in developing countries, and such circumstances may get worse in the context of rapid urbanization. Approximately half of urban slums in India are non-notified (i.e., not recognized by the government), making it extremely difficult, if not impossible, to access formal municipal water supplies [9]. In the absence of provision of water by the government, informal distribution systems, like the one we have described, arise out of necessity. Provision of water is taken over by water vendors (who in KB’s case are residents of the community) or other suppliers, with resulting deterioration in water-related health and social equity indicators.

In circumstances where household storage of water is unavoidable, our water quality findings suggest a beneficial role for household-level interventions, including provision of narrow-mouthed safe storage containers and chlorination. More crucially, our findings regarding point-of-source quality, quantity of use, and cost of water highlight the need for equitable access to the municipal water supply to improve the water situation in KB. Since KB’s lack of formal water access arises from its legal status as a non-notified slum, transformation of this situation will not happen without confronting larger questions of social exclusion, social justice, and urban governance. The critical importance of connecting communities such as KB to the formal municipal water supply cannot be overstated. Indeed, this may be the only way to ensure that residents of marginalized slums can attain universally accepted minimum health and social equity standards for this most fundamental of human needs.

## Abbreviations

BNA: Baseline needs assessment; INR: Indian rupees; KB: Kaula Bandar; l/c/d: Liters per capita per day; PUKAR: Partners for urban knowledge action, and research; SWA: Seasonal water assessment; WHO: World health organization

## Competing interests

The authors declare that they have no competing interests.

## Authors’ contributions

RS made contributions to the study design, data collection, interpretation of data, and drafting of the manuscript. SS, TS, and KS contributed to the study design, data collection, interpretation of the data, and critical revision of the manuscript. JO contributed to the study design, statistical analysis, interpretation of data, and critical revision of the manuscript. DEB contributed to study design, interpretation of data, and critical revision of the manuscript. APD contributed to study design, data collection, interpretation of data, and critical revision of the manuscript. All authors read and approved the final paper.

## Pre-publication history

The pre-publication history for this paper can be accessed here:

http://www.biomedcentral.com/1471-2458/13/173/prepub
